# TNF-α and IL-8 levels are positively correlated with hypobaric hypoxic pulmonary hypertension and pulmonary vascular remodeling in rats

**DOI:** 10.1515/biol-2022-0650

**Published:** 2023-07-27

**Authors:** Haixia Shi, Yongfeng Zhao, Su Li, Haitao Wu, Dehua Ma, Chenchen Wan

**Affiliations:** Department of Emergency, Affiliated Hospital of Qinghai University, Xining City, Qinghai Province 810001, China

**Keywords:** pulmonary arterial hypertension, TNF-α, mean pulmonary arterial pressure, pulmonary vascular remodeling

## Abstract

The expression status of proinflammatory cytokines in high-altitude pulmonary arterial hypertension (PAH) has been well studied. However, the changes in interleukin (IL)-8 and tumor necrosis factor α (TNF-α) during the reversible changes in pulmonary vascular remodeling (PVR) in PAH after detaching from a hypobaric hypoxic environment have not been elucidated. This investigation elucidated a high-altitude PAH rat model. Then, PAH rats in the high-altitude group were maintained in the high-altitude area, and rats in the low-altitude group returned to the low-altitude area. After 0, 10, 20, and 30 days of PAH modeling, right ventricular systolic pressure (RVSP) and the mean pulmonary arterial pressure (mPAP) were assessed. Right ventricular (RV) hypertrophy was reflected by the ratio of RV/[left ventricle + interventricular septum (S)]. Pathological changes in PVR were accessed by hematoxylin-eosin staining, and medial wall thickness (WT%) and medial wall area (WA%) were measured. TNF-α and IL-8 levels in pulmonary artery tissues and blood were measured with Western blot assay and enzyme-linked immunosorbent assay, respectively. Our results showed that PAH rats exhibited a substantial increase in RVSP and mPAP, RV hypertrophy, PVR, and enhanced generation of TNF-α and IL-8. Then, we found that these pathological changes were gradually aggravated and TNF-α and IL-8 levels were increased in rats in the high-altitude group after 10, 20, and 30 days of PAH modeling. In contrast, the mPAP was decreased and PVR was alleviated in rats in the low-altitude group, accompanying with reduced TNF-α and IL-8 production. In conclusion, our study demonstrated that the generation of TNF-α and IL-8 was also reversible during the reversible changes in PVR after detaching from a hypobaric hypoxic environment. Thus, proinflammatory cytokine TNF-α and IL-8 levels are positively correlated with PVR severity.

## 1 Introduction

Pulmonary arterial hypertension (PAH) is a rare and extremely harmful cardiovascular disorder, which is considered cancer in the cardiovascular system [1]. PAH is classified by vascular stenosis and increased resistance in small pulmonary arteries, eventually causing right ventricular (RV) hypertrophy, afterload, and failure [2]. It is generally known that adverse pulmonary vascular remodeling (PVR) is a critical pathological feature contributed to the increased vascular resistance in PAH, which is a complex pathophysiological process manifested by pulmonary vascular proliferation, thrombosis inflammation, and fibrosis [3,4]. So far, multifarious advanced pharmacological therapies have been developed for the PAH treatment. However, the long-term prognosis of PAH patients is still unsatisfactory [5,6]. Therefore, the underlying mechanisms of PVR during PAH have not been well understood.

Qinghai province in China is a high-altitude region with an average elevation of above 3,000 m. It has a special climate with low pressure, low oxygen, high radiation, and high cold environments, which is significantly different from low altitude and plain regions. It was found that individuals with chronic obstructive pulmonary disease who have been residing at high-altitude areas for a prolonged time had higher morbidity and mortality than those living at lower altitude or plain region [7]. Therefore, it is necessary to explore the mechanisms of PVR in high-altitude PAH. It is generally known that inflammatory cells play an essential role in hypoxic PVR. Multiple advanced research has proved that a variety of inflammatory cells and proinflammatory cytokines are involved in the process of hypoxic PVR [8,9,10]. Interleukin (IL)-8 is an inflammatory cytokine and also a chemokine that is mainly produced by monocyte phagocytes and endothelial cells. Epithelial cells and fibroblasts can also generate IL-8 under appropriate stimulation conditions. IL-8 could stimulate the chemotaxis of neutrophils T cells, lymphocytes, and eosinophils and then damage endothelial cells, leading to microcirculation congestion and tissue necrosis [11]. Tumor necrosis factor α (TNF-α) is a proinflammatory cytokine that can potently regulate the pulmonary circulation [12]. It could rapidly activate neutrophils to release ILs and induce adhesion molecule expression, resulting in neutrophil aggregation and microvascular obstruction in ischemic area, and finally cause vascular endothelium damage [13]. So far, the status and significance of TNF-α and IL-8 in hypoxic PVR have been commonly studied. However, there are no basic and clinical studies at home and abroad on the changes in TNF-α and IL-8 during the reversible changes in PVR after detaching from a hypobaric hypoxic environment, and the histopathological basis is missing.

In this study, a model of high-altitude PAH in Wistar rats was established, and the rats returned to the low-altitude area after PAH modeling. We investigated the expression changes in proinflammatory cytokines TNF-α and IL-8 during the reversible changes in PVR after returning to the low-altitude area and analyzed the relationship between proinflammatory cytokine production and the severity of PVR.

## 2 Materials and methods

### 2.1 Animal experiments

The standard clean-grade healthy male Wistar rats (6–7 weeks; 170–200 g) utilized in this research were supplied by Beijing HFK Bioscience Co., Ltd (Beijing, China) and were kept in a standard SPF-grade cage, in standard housing conditions (12 h cycle of dark/light, temperature = 22–25°C, humidity = 55–60%) with ad libitum food and water. Wistar rats were categorized into three groups via the random number table method: control, high altitude, and low altitude (*n* = 40 per group). First, a model of high-altitude PAH in Wistar rats was established by simulating a 5,000 m altitude environment with a hypobaric oxygen chamber provided by the High Altitude Medical Research Center of Qinghai University [14]. The pulmonary artery pressure was assessed to determine the successful establishment of PAH model. Then, Wistar rats in the high-altitude group were maintained in the high-altitude area (Qinghai province with an average elevation of above 3,000 m) after successful modeling, while rats in the low-altitude group were returned to the low-altitude area (plain region with elevation less than 200 m) after PAH modeling. And rats in the healthy control group were maintained in the low-altitude area without PAH modeling and any treatment. Rats in different groups have the same feeding, housing, and environmental conditions except for different high and low altitudes. Each rat was evaluated by independent and well-trained investigators blinded to the experimental grouping. The subsequent experiments were conducted after 0, 10, 20, and 30 days of modeling. The animal protocols were authenticated by the Ethics Committee of Affiliated Hospital of Qinghai University.


**Ethical approval:** The research related to animal use has been complied with all the relevant national regulations and institutional policies for the care and use of animals, and has been approved by the Ethics Committee of Affiliated Hospital of Qinghai University.

### 2.2 Hemodynamic Evaluation

Hemodynamic parameters were evaluated as described previously [15,16]. First, to anesthetize, 20% urethane (1.0 g/kg) was injected intraperitoneally until rats are fully relaxed, unresponsive to pain stimulation, and have normal breathing and heart rate, and the anesthesia was maintained for approximately 2 h. The rats were then fixed to the operating table. The pulmonary artery pressure was identified by the right heart catheterization method, where a polyethylene catheter was administered into the pulmonary artery and RV via the right external jugular vein. At the other end of the catheter, a P50 pressure transducer was connected to a biological function experimental system (BL-420; Tai Meng Technology, Chengdu, China), and the inserted catheters’ position was correctly calibrated by waveform depicted on the biological function experimental system. The mean pulmonary arterial pressure (mPAP) and right ventricular systolic pressure (RVSP) were assessed and recorded.

### 2.3 RV hypertrophy evaluation

After hemodynamic evaluation, the rat was euthanized by exsanguination after being anesthetized with 2% isoflurane inhalation. The rat heart was exposed and filled with 0.9% saline. The left ventricle (LV) and a tiny hole in the right atrium were punctured with a needle to continuously infuse for 30 min to remove remnant blood. Next, the atrial tissues were cut off. The LV, interventricular septum (S), and RV were separated along the edge of the interventricular septum. After absorbing the moisture with filter paper, the RV, LV, and S were weighed. The degree of RV hypertrophy index was reflected by the ratio of RV/[LV + S].

### 2.4 HE staining

The lungs were collected for histological evaluation after euthanasia. Pulmonary vessels at a distance of 2 mm from the pulmonary hilum and with 20–150 µm external diameter were randomly selected for assessment. Lung tissues from the pulmonary hilum were then fixed in 10% formalin overnight, followed by paraffin embedding. Then, 5-µm‐thick tissue slices were performed, deparaffinized with xylene, and dehydrated with ethanol dilution series. Next, hematoxylin was utilized for tissue staining for 8 min and then stained the cytoplasm in eosin (Nanjing Yulu experimental equipment Co., Ltd, Nanchang, China). Finally, the slices were dehydrated, mounted in xylene solution for transparency, and sealed with neutral resin. A light microscopy (magnification 200×; BX51; Olympus, Japan) was used for histological evaluation. In addition, six small pulmonary arteries per rat were selected, and the wall thickness (WT), lumen area (LA), vascular area (VA), and external diameter (ED) were calculated by the ImageJ 1.43 software (National Institutes of Health, Bethesda, MD, USA). The percentage of medial WT% was calculated by WT = (2 × WT/ED × 100%), and the percentage of medial wall area (WA) was calculated by WA = ([VA–LA]/VA × 100%).

### 2.5 Western blot analysis

Whole proteins from rat pulmonary arteries were extracted using lysis buffer, and their concentration was accessed by the bicinchoninic acid method. The same amounts of samples (30 µg/lane) were isolated via sodium dodecyl sulfate-polyacrylamide gel electrophoresis at 70 V for 30 min and then 120 V for 90 min and transplanted onto PVDF membranes, which were then incubated at 4°C in primary antibody (Abcam, UK) overnight, and then for 2 h in secondary antibody (1:2,000, Abcam, ab6721) at 37°C. The membranes were visualized via an ECL kit (Thermo Fisher, Waltham, MA, USA), and the gray intensities were determined via the ImageJ software. The protein levels were normalized to GAPDH. The primary antibodies were listed as follows: anti-IL-8 antibody (1:1,000, ab235584), anti-TNF-α antibody (1:1,000, ab183218), and anti-GAPDH antibody (1:2,500, ab9485).

### 2.6 Enzyme-linked immunosorbent assay (ELISA)

Blood samples (1 mL) were obtained from the rat heart and placed in a centrifuge tube (1.5 mL). Then, serum was extracted by 10 min centrifuging blood at 3,000 rpm and 4℃. The serum TNF-α level was tested with TNF-α Detection ELISA Kit (Boster Biological Technology, Wuhan, China) and the serum IL-8 level was detected with IL-8 Detection ELISA Kit (Jianglai Biological Technology, Shanghai, China) as per manufacturer’s guide.

### 2.7 Statistical measurements

All quantitative data were obtained from at least triplicate experiments and represented as mean ± standard error of mean (SEM). Data analysis was performed on SPSS 22.0 software. Normally distributed data were assessed by the Shapiro–Wilk test. Levene’s test verified the homogeneity of variances. One-way analysis of variance followed by post hoc analysis (Tukey–Kramer correction) was used for the normally distributed data and homogeneous variances. For non-normally distributed data or when the variances were not homogeneous, Kruskal–Wallis non-parametric test was applied. Significance was deemed at *P  <* 0.05.

## 3 Results

### 3.1 The mPAP and RVSP of high-altitude PAH rats were gradually decreased after returning to low altitude

**Figure 1:**
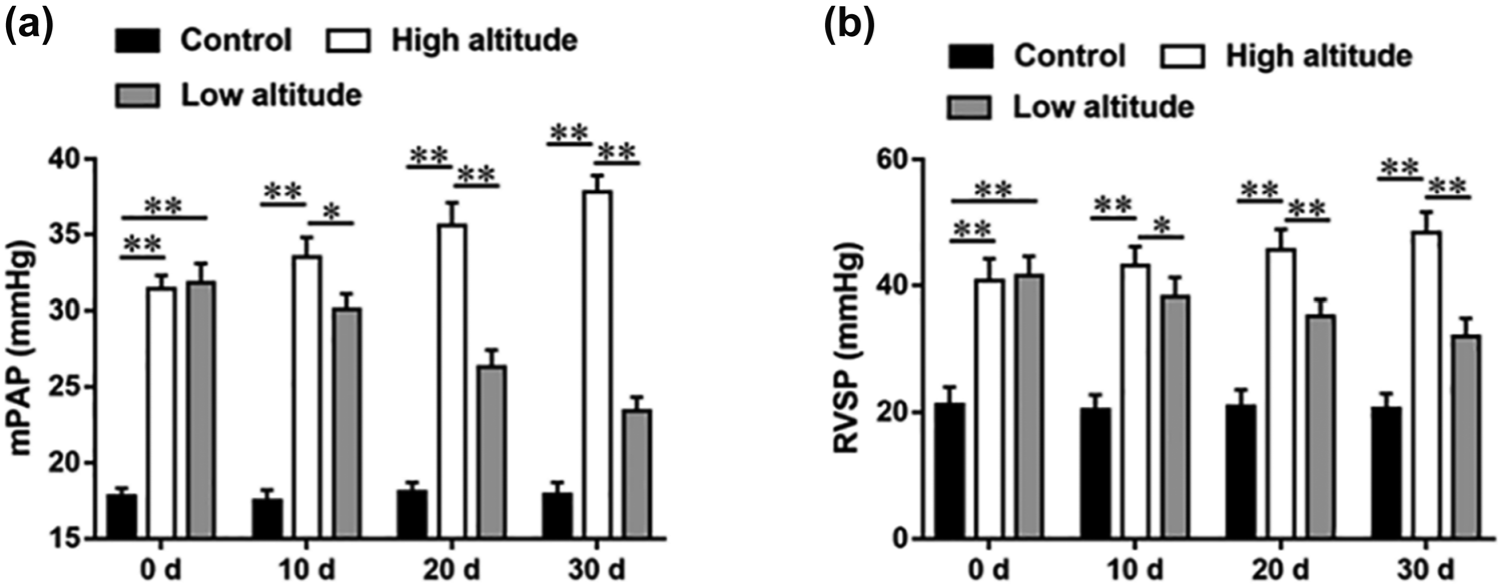
The mPAP and RVSP of rats in each group after PAH modeling of 0, 10, 20, and 30 days. Wistar rats were randomly divided into three groups: control, high altitude, and low altitude (*n* = 40 per group). A model of high-altitude PAH in Wistar rats was established. Rats in the high-altitude group were maintained in the high-altitude area rats in the low-altitude group were returned to the low-altitude area after successful modeling. And rats in the healthy control group were maintained in the low-altitude area without any treatment. (a) The mPAP of rats in each group was accessed after PAH modeling of 0, 10, 20, and 30 days. (b) The RVSP of rats in each group were accessed after PAH modeling of 0, 10, 20, and 30 days. Data from at least triplicate experiments were presented as mean ± SEM. *P < 0.05, **P < 0.01.

A model of high-altitude PAH in Wistar rats was established. Then, Wistar rats in the high-altitude group were maintained in the high-altitude area after successful modeling, while rats in the low-altitude group were returned to the low-altitude area after PAH modeling. As shown in Figure 1a, after PAH modeling, the mPAP of high-altitude rats and low-altitude rats were prominently elevated compared with rats in the control group, indicating that the PAH model was successfully established. Then, we found that the mPAP of high-altitude PAH rats was gradually increased after 10, 20, and 30 days of PAH modeling, whereas the mPAP of PAH rats was gradually decreased after returning to low altitude. Moreover, the RVSP of rats showed the same trend as mPAP. The RVSP of high-altitude PAH rats was increased after 10, 20, and 30 days of PAH modeling, while the RVSP of low-altitude PAH rats was gradually decreased after 10, 20, and 30 days of PAH modeling (Figure 1b).

### 3.2 RV hypertrophy of high-altitude PAH rats was alleviated after returning to low altitude

**Figure 2:**
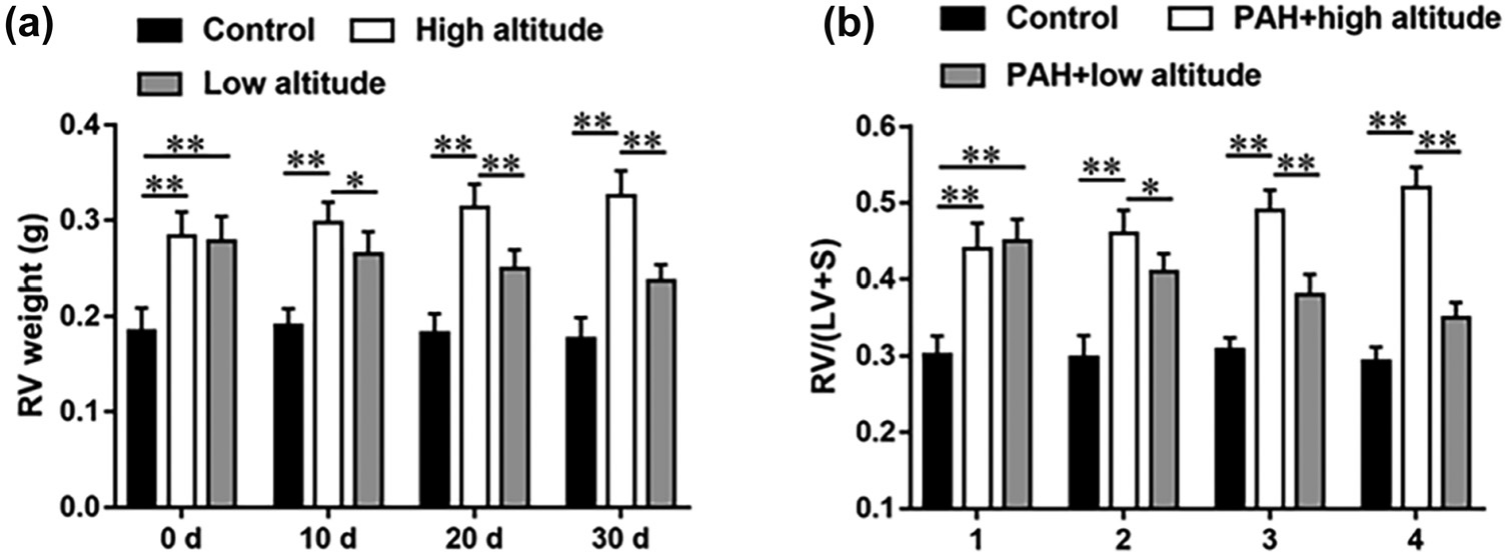
RV hypertrophy of rats in each group after PAH modeling of 0, 10, 20, and 30 days. (a) RV weight of rats in each group was accessed after PAH modeling of 0, 10, 20, and 30 days. (b) The weight ratio of (RV/LV + S) of rats in each group was accessed after PAH modeling of 0, 10, 20, and 30 days. Data from at least triplicate experiments were presented as mean ± SEM. *P < 0.05, **P < 0.01.

As shown in Figure 2a and b, after PAH modeling, the indices of RV hypertrophy, such as RV weight and RV/LV + S, were substantially higher in high-altitude and low-altitude PAH rats compared with the control group. The RV weight and RV/LV + S weight ratio of PAH rats also enhanced in the high-altitude group after 10, 20, and 30 days of PAH modeling, whereas the low-altitude PAH rats showed reduced RV weight and RV/LV + S weight ratio after returning to low altitude, indicating that the RV hypertrophy of PAH rats was alleviated after returning to low altitude.

### 3.3 The PVR of high-altitude PAH rats was alleviated after returning to low altitude

**Figure 3:**
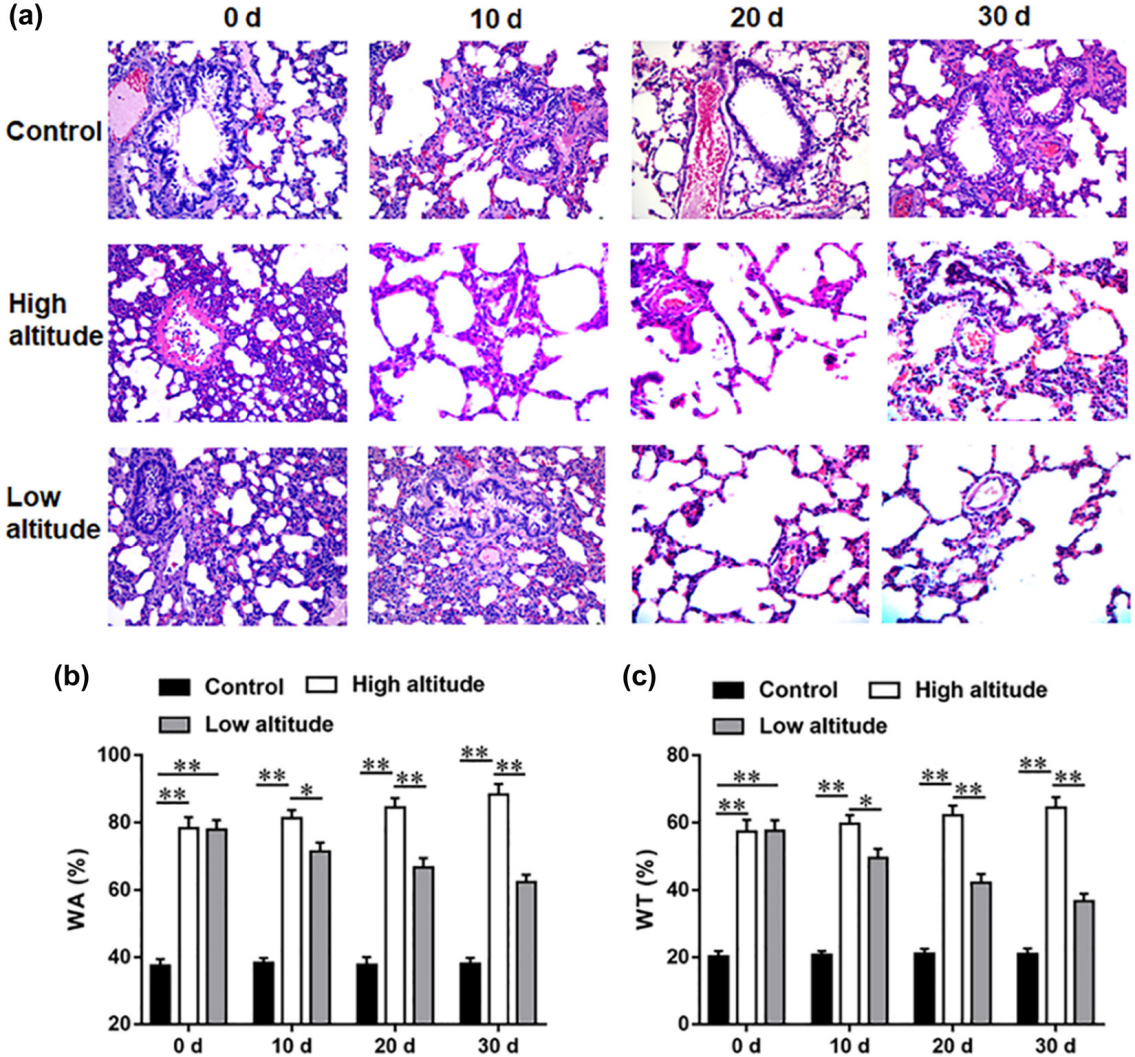
The pulmonary vascular remodeling of rats in each group after PAH modeling of 0, 10, 20, and 30 days. (a) HE staining showed the severity of pulmonary vascular remodeling of rats in each group after PAH modeling of 0, 10, 20, and 30 days. (b and c) WA% and WT% of rats in each group were calculated after PAH modeling of 0, 10, 20, and 30 days. Data from at least triplicate experiments were presented as mean ± SEM. *P < 0.05, **P < 0.01.

Pulmonary arteriole remodeling was among the most critical PAH pathological characteristics. We then evaluated the changes in hypoxic pulmonary arteriole remodeling in high-altitude PAH rats after returning to low-altitude environment. Hematoxylin-eosin (HE) staining revealed that the control group exhibited the thin and continuous arteriole wall. However, pulmonary arterial wall thickening and lumen diameter reduction and smooth muscle cell proliferation in the middle layer were observed in the PAH rat model. Moreover, we observed that pulmonary arterial wall thickening and pulmonary arteriole remodeling in high-altitude PAH rats was gradually serious after 10, 20, and 30 days of PAH modeling, and these pathological features were alleviated after returning to low altitude (Figure 3a). As displayed in Figure 3b and c, WT% and WA% were conspicuously elevated in the PAH rat model in comparison to the control group. Compared with day 0 of modeling, WA% and WT% were gradually elevated in high-altitude rats and reduced in low-altitude PAH rats after 10, 20, and 30 days of PAH modeling. These results emphasized that the PVR of PAH rats was alleviated after returning to low altitude.

### 3.4 The levels of TNF-α and IL-8 of high-altitude PAH rats were reduced after returning to low altitude

**Figure 4:**
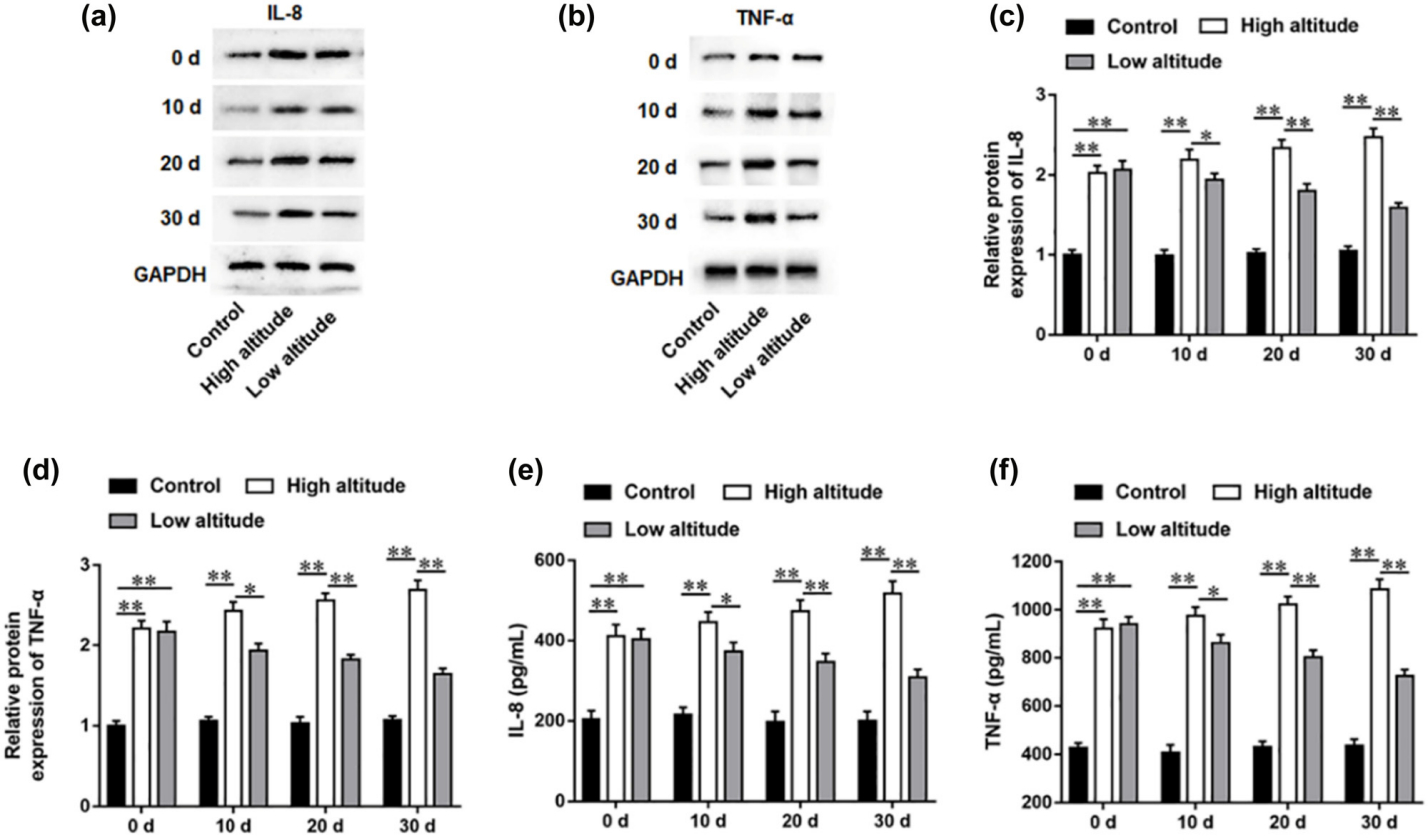
The levels of TNF-α and IL-8 of rats in each group after PAH modeling of 0, 10, 20, and 30 days. (a–d) The levels of TNF-α and IL-8 in pulmonary artery tissues of rats in each group were accessed after PAH modeling of 0, 10, 20, and 30 days. (e and f) The levels of TNF-α and IL-8 in blood samples of rats in each group were accessed after PAH modeling of 0, 10, 20, and 30 days. Data from at least triplicate experiments were presented as mean ± SEM. *P < 0.05, **P < 0.01.

It is generally known that inflammatory cytokines function in hypoxic PVR. To find the relationship between inflammatory cytokines and the severity of PVR, we investigate the changes in TNF-α and IL-8 levels in PVR in high-altitude PAH rats after returning to low-altitude environment. Our results illustrated that TNF-α and IL-8 levels in pulmonary artery tissues were prominently elevated after PAH modeling. Furthermore, it was suggested that TNF-α and IL-8 levels in pulmonary arteries of high-altitude PAH rats were gradually increased after 10, 20, and 30 days of PAH modeling, whereas TNF-α and IL-8 levels in rats were gradually reduced after returning to low altitude (Figure 4a–d). Likewise, ELISA results showed that TNF-α and IL-8 levels in blood samples exhibited the same expression trend as that in pulmonary artery tissues (Figure 4e and f).

## 4 Discussion

Hypoxic PVR is a complex network in which multiple cells and molecules participate and influence each other. A growing number of studies have illustrated that a variety of inflammatory cells, proinflammatory cytokines, chemical mediators, and adhesion molecules are linked with the process of hypoxic PVR [8,9,10]. Hypoxia facilitated the production of proinflammatory cytokines and the inflammatory cells’ infiltration into the vascular adventitia in lung tissues, which is a critical pathogenesis contributing to PVR [17]. Endothelial cell injury is the pivotal starting point in vascular inflammatory lesions, which causes the damage of vascular endothelial barrier. After that, circulating inflammatory cytokines can directly endanger the vascular wall. Meanwhile, hypoxia can directly induce the transcriptional activation of cell adhesion molecules in endothelial cells and enhance the interaction of vascular endothelial cells and circulating inflammatory cells, Finally, the proinflammatory microenvironment formed around the pulmonary vessels increases the expression of inflammatory factors and eventually accelerates pulmonary vasoconstriction and PVR [8]. The severity of PVR is positively correlated with the thickness of the vessel wall.

**Figure 5:**
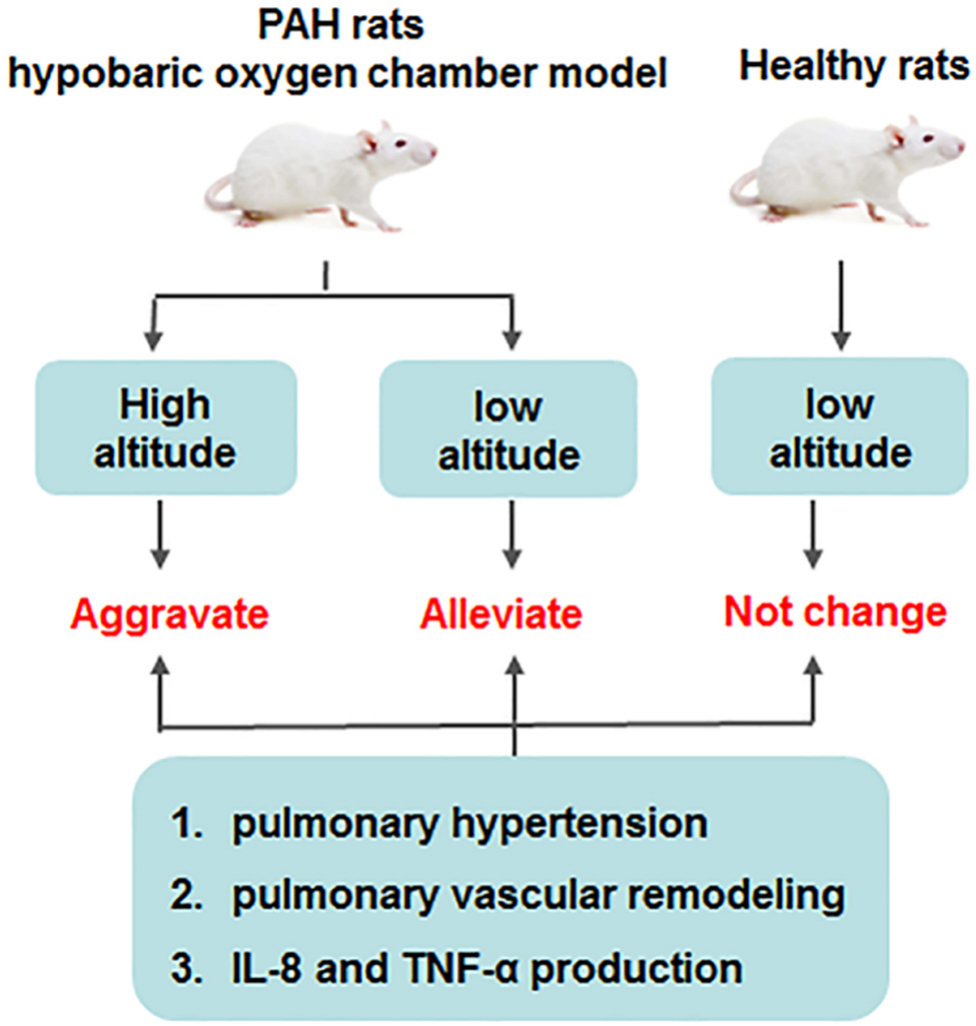
The production of TNF-α and IL-8 was reversible during the reversible changes of pulmonary vascular remodeling after detaching from a hypobaric hypoxic environment. TNF-α and IL-8 levels are positively correlated with hypobaric hypoxic pulmonary hypertension and pulmonary vascular remodeling in rats.

The proinflammatory cytokines, such as IL-1β, TNF-α, IL-6, and IL-8, were shown to be excessively produced in PAH patients’ lung tissues, which contributed to PVR and was related to disease severity [18,19]. Furthermore, it was illustrated that TNF-α could enhance pulmonary vascular reactivity, reduces prostaglandins synthesis in the smooth muscle cells of pulmonary artery, and induce pulmonary vasoconstriction [20]. A previous study implicated that TNF-α upregulation was indicated in the PAH rat model and hypoxia-mediated pulmonary artery smooth muscle cells, which is defined as an essential regulator in PAH pathology [21]. Moreover, the plasma TNF-α concentration was prominently elevated in the chronic thromboembolic PAH rat model, and TNF-α protein level in pulmonary artery was positively correlated with mPAP and WA% [22]. In addition, the current study has proved that TNF-α and IL-8 expressions were markedly increased in the plasma samples of PAH patients compared with healthy controls [23]. Meanwhile, it was found that the PAH rat model exhibited increased RVSP, thickened pulmonary vessel wall, and RV hypertrophy, which was accompanied by upregulated IL-8 expression in the pulmonary artery endothelial cells [24]. More notably, it was demonstrated that persistent hypoxia induced PAH and enhanced PAP, lung edema, RV hypertrophy, lung vascular proliferation, and the secretion of inflammatory cytokines IL-8, TNF-α, and IL-6 in lung tissues of rats [25]. These results are consistent with our results that the secretion of proinflammatory cytokines TNF-α and IL-8 was positively correlated with the severity of PVR (Figure 5).

At present, the changes and significance of TNF-α and IL-8 in hypoxia-induced PAH and hypoxic PVR have been well studied. However, the changes in TNF-α and IL-8 during the reversible changes in PVR after detaching from a hypobaric hypoxic environment have not been elucidated. This investigation successfully established a hypobaric hypoxia-triggered high-altitude PAH rat model. High-altitude PAH rats exhibited a substantial increase in RVSP and mPAP, RV hypertrophy, pulmonary arterial wall thickening, lumen diameter reduction, and proliferation of smooth muscle cell, and the increased production of TNF-α and IL-8 in pulmonary artery tissues. The data of this investigation were consistent with the previous research, which demonstrated that high-altitude PAH exhibited elevation of inflammatory cell infiltration, cytokine levels (TNF-α IL-6, and IL-1β), and chemokine levels. The above information highlighted the importance of the crosstalk between hypoxia and inflammation [26]. Then, our results illustrated that these pathological changes were gradually aggravated in the rats maintained in the high-altitude area after 10, 20, and 30 days of PAH modeling. In contrast, for rats returned to the low-altitude region, the mPAP was decreased, and RV hypertrophy and PVR were alleviated after 10, 20, and 30 days of PAH modeling. Meanwhile, TNF-α and IL-8 levels in pulmonary artery tissues and blood samples were also gradually reduced during the reversible changes of PVR.

In conclusion, our study demonstrated that the levels of proinflammatory cytokines TNF-α and IL-8 increased with the severity of hypobaric hypoxic PVR The production of TNF-α and IL-8 was also reversible during the reversible changes in PVR after detaching from a hypobaric hypoxic environment. Thus, proinflammatory cytokines TNF-α and IL-8 levels are positively correlated with the severity of PVR. Our study provides a pathological basis for the involvement of proinflammatory cytokines in the pathogenesis of PVR in hypobaric hypoxic PAH.

Our results still have potential limitations and confounding or biased factors, such as small sample size of experimental animals, genetic factors (different litters of rats), and environmental factors (animals raised in different regions, presence of noise and different feeder behavior, etc.). In future research, we will expand the sample size of experimental animals and strive to eliminate these potential confounding or bias factors to make our research results more convincing.
